# Floss Silk a Vehicle in Fang Filling

**Published:** 1872-07

**Authors:** B. M. Wilkerson


### ARTICLE III.

### *Floss Silk a Vehicle in Fang Filling.*

BY B. M. WILKERSON, D.D.S.

To those who are not well pleased with their fang fillings I would say: Entangle a piece of white floss silk into a very small pellet, saturate it in liquid gutta percha or oxychloride of zinc—prepared as ordinary for filling, but slow to set—carry it well up to the apex of the root and continue to fill the fang with same material. Use the same care in preparing as you would for gold. Both give me better satisfaction than gold and my patients perfect comfort.

